# The Antioxidant Properties of Alfalfa (*Medicago sativa* L.) and Its Biochemical, Antioxidant, Anti-Inflammatory, and Pathological Effects on Nicotine-Induced Oxidative Stress in the Rat Liver

**DOI:** 10.1155/2022/2691577

**Published:** 2022-03-26

**Authors:** Mahdieh Raeeszadeh, Javad Beheshtipour, Rozhin Jamali, Abolfazl Akbari

**Affiliations:** ^1^Department of Basic Sciences, Sanandaj Branch, Islamic Azad University, Sanandaj, Iran; ^2^Young Researchers and Elite Club, Sanandaj Branch, Islamic Azad University, Sanandaj, Iran; ^3^Department of Physiology, School of Veterinary Medicine, Shiraz University, Shiraz, Iran

## Abstract

*Medicago sativa* Linn or alfalfa is a tonic plant rich in proteins, vitamins, and minerals that is used to treat many diseases due to its pharmacological properties such as anti-inflammatory and antioxidant activities. So, the aim of this study was to evaluate the efficacy of alfalfa methanolic extract (AME) on the prevention of liver damage caused by nicotine. The total phenols, flavonoids levels, and the free radical scavenging activity of its extract (IC50) were measured. In this study, 30 Wistar rats were randomly divided into 5 groups as control (untreated), N (nicotine only), T1, T2, and T3 (nicotine + AME 100, 250, and 500 mg/kg/day, respectively). AME (orally) and nicotine (intraperitoneal injection, 0.5 mg/kg/day) were then administered for 21 days. Weight gain, the liver-to-body weight ratio, liver functional enzymes, and the lipid profile were measured. Moreover, we evaluated oxidative stress, proinflammatory parameters, and histopathological changes in the liver. Total phenols, flavonoids, and IC50 were determined as 51.68 ± 0.62 mg GAE/g, 18.55 ± 1.01 mg QE/g, and 350.91 ± 16.46 *μ*g/ml, respectively. Nicotine changed the measured parameters to abnormal. AME increased weight gain, the liver-to-body weight ratio, and enzymatic antioxidant levels and decreased malondialdehyde, liver functional enzymes, and proinflammatory cytokine levels. The lipid profile and histopathological changes have also been improved by AME in a dose-dependent manner. The results showed that AME in a dose-dependent manner by improving the inflammation and oxidative damage could improve the liver damage caused by nicotine.

## 1. Introduction

Nicotine (C_10_H_14_N_2_) is the main alkaloid of tobacco and an addictive substance in cigarettes [1]. Nicotine could increase the risks of liver failure, cardiovascular diseases, type 2 diabetes, pulmonary disorders, several types of cancers, and infertility [2, 3]. In addition, it can be easily absorbed through the skin and respiratory tract. Nicotine can also reach peak levels in the bloodstream [4]. Notably, in the human liver, the cytochrome P450 2A6 (CYP2A6) plays a pivotal role in nicotine metabolism, and it converts approximately 80% of the absorbed nicotine into cotinine [5]. Some studies performed on humans and rodents have shown that nicotine metabolism by cytochrome P450 enzymes produces reactive oxygen species (ROS) or free radicals, which can defect the antioxidant defense system and enhance lipid peroxidation of polyunsaturated fatty acids (PUFAs). Finally, this process consequently induces oxidative stress in body tissues, especially in the liver, because the main metabolism center of nicotine is the liver [6]. In the end, the obtained product of lipid peroxidation is an active and highly reactive aldehyde compound named as malondialdehyde (MDA), which can be used as an indicator to evaluate oxidative stress [7].

Many organisms utilize an antioxidant defense system including free radical scavenging, various enzymatic and nonenzymatic antioxidants, and metal-chelating agents to counteract the oxidative damage resulting from ROS [8]. Notably, many medicinal plants and foods (such as vegetables, fruits, and grains) are rich in natural antioxidants [9]. Moreover, phytoestrogenic compounds and various kinds of vitamins are well-known antioxidants found in many plants [10, 11]. In this regard, *Medicago sativa* (alfalfa) is one of the herbs rich in phytoestrogenic compounds such as apigenin, luteolin, coumestrol, quercetin, medicarpin, daidzein, and genistein as well as various vitamins, especially vitamin C [12]. Besides the antioxidant role of alfalfa, its beneficial effects on some diseases such as diabetes, thalassemia, various cancers, renal disorders, and hypercholesterolemia have been also identified. Considering the abovementioned beneficial characteristics, this plant is processed and considered in various medicinal forms worldwide [13].

Nowadays, with the increasing trend of risk factors of human diseases and people's different attitudes in different countries, the global trend towards the treatment of various diseases has led to the use of medicinal plants and natural foods [14–16]. Therefore, performing comprehensive studies is essential to investigate the role of plants and natural compounds when facing toxic compounds as well as studying the response of metabolic centers (especially the liver) to this process. Hence, in this work, we aimed to evaluate the efficacy of alfalfa methanolic extract (AME) on controlling nicotine-induced oxidative stress in the liver of male Wistar rats.

## 2. Materials and Methods

### 2.1. Plant Material

Fresh alfalfa was collected (the month of June, 5 kg) from the fields of Qorveh city, located in western Iran, with latitude 47°44′38.768^″^E and 35°11′57.820^″^N. The plant was then cultivated, and its seeds were similar throughout the region because they were provided by the local agriculture organization. The identity of this plant (No. 1856) was confirmed by the Herbarium Center of the University of Kurdistan.

### 2.2. Preparation of Alfalfa Methanolic Extract (AME)

The plant was milled after drying it at 25°C. As demonstrated in our earlier works, the IC50 of the methanolic extract is lower than that of the ethanolic one while the concentration of polyphenols and flavonoids isolated in the methanolic extract is higher than those in the ethanolic extract [17]. Moreover, the separation of polar and nonpolar phytochemical compounds within a wide range can be achieved more feasibly via maceration methods by methanol [18]. Hence, in this study, the methanolic solvent was used. Thereafter, 200 g of alfalfa powder was soaked in 2 liters of methanol for 5 days. Subsequently, the resulting mixture was smoothed with Whatman No. 1 filter paper and then centrifuged at 3000 g for 15 minutes. In the following, the supernatant was concentrated by the connected rotary to the vacuum pump at 45°C. Next, the methanol was removed under negative pressure. Then, the extract was obtained in the form of powder and stored at -4°C [19]. 100, 250, and 500 mg/kg concentrations of the extract were prepared by sterile distilled water daily to maintain stability. The fresh extract was orally administered (P.O.) via a gastric tube daily [20]. The extract is prepared and used daily in distilled water to maintain stability.

### 2.3. Determination of Total Phenolic Content

The amounts of total phenolic compounds were evaluated using the Folin-Ciocalteu reagent in the extract [21]. Briefly, the Folin-Ciocalteu reagent (100 *μ*l) and distilled water (1.2 ml) were added to each test tube containing 20 *μ*l of alfalfa extract (concentration, 1 mg/ml). After 10 minutes of storage at room temperature, 300 *μ*l of the Na_2_CO_3_ solution (20% *w*/*v*) was added to the tubes. Following 120 min storage in darkness, their absorbance level was measured at 765 nm. Afterward, gallic acid was used to draw the calibration curve (absorbance = 0.0104 gallic acid *μ*g − 0.0068, R2 = 0.9936). The obtained results were then expressed as gallic acid equivalents (GAE) in mg/g of the extract.

### 2.4. Determination of Total Flavonoid Content

The colorimetric method of aluminum chloride was used to measure the amount of total flavonoid [22]. According to this method, 0.5 ml of extract (1 mg/ml) was added to the test tubes. Thereafter, 1.5 ml of 95% ethanol, 0.1 ml of 10% aluminum chloride, 0.1 ml of 1 mM potassium acetate, and 2.8 ml of distilled water were added to each tube and then mixed with the extract, respectively. After 30 min storage at 25°C, the absorbance level of the mixtures was measured at 415 nm. Subsequently, the standard curve was plotted with quercetin in this work (absorbance = 0.0165 *x* + 0.0309, R2 = 0.991). The total flavonoid content was then expressed as mg quercetin equivalent (QE)/g of the extract [23].

### 2.5. Determination of Free Radical Scavenging Activity

The free radical scavenging activity of the extract was measured using DPPH (2,2-diphenyl-1-picrylhydrazyl) radicals [24]. Accordingly, different concentrations (10, 20, 50, 100, 200, 500, and 1000 *μ*g/ml) of the obtained extract were prepared in methanol solvent. Afterward, for each concentration, 300 microliters of the extract was mixed with 2.7 ml of DPPH methanol solution (0.06 mM) and it was then well-shaken. All the mixtures were incubated for 30 min in darkness until stabilization of the reaction. After this period, the absorbance level of the mixtures was read at 517 nm. During this process, vitamin C was considered the control group. Also, the following formula was used to calculate the inhibition percent of DPPH radicals by the extract: %of DPPH radical scavenging = [(Ac − As)/Ac] × 100, where Ac is the absorption of DPPH solution and As is the absorption of extract or vitamin C.

IC50 was determined as the concentration of the extract that inhibited 50% of DPPH radicals. Accordingly, it was calculated from the graph of the inhibition percentage against extract concentration. Notably, the assay was performed in triplicate.

### 2.6. Acute Toxicity (LD50) Study

The toxicity tests were carried out according to the Organization for Economic Cooperation and Development (OECD) test guideline, i.e., OECD Guideline 423 used for the acute oral toxicity test. Before the start of the experiment, the body weight of animals was recorded individually for calculating the proper treatment dosage. To determine the LD50 of AME, 12 Wistar rats (200-250 g) were randomly divided into 4 groups (3 rats per group), and 5, 50, 300, and 2000 mg extract/kg body weight, P.O., were separately administered for each group, respectively. Signs of toxicity and possible death of animals were monitored for 24 hours to calculate the median lethal dose (LD50) of alfalfa. All animals were observed at least once during the first 30 min in the first 24 hours with great consideration given for the first 4 hours following vehicle or alfalfa methanol extract administration, and then they were monitored once a day for 14 days. This observation was done to check the onset of clinical or toxicological symptoms according to the OECD guideline. All observations included changes in the skin and fur, eyes, and mucous membranes, and behavioral patterns were systematically recorded and maintained with an individual record. In addition, consideration was given for observations of convulsions, tremors, diarrhea, salivation, lethargy, sleep, coma, and mortality. At the end of the study, all the animals in all the dose groups were sacrificed and the internal organ-body was compared with values from the control group [25].

### 2.7. Nicotine Preparation

In this study, a 2% solution of nicotine hydrogen tartrate salt was prepared by sterile distilled water, which was then used for intraperitoneal injection (I.P.) at the dose of 0.5 mg/kg [26].

### 2.8. Animals

For this study, 30 male Wistar rats (200-250 g, 6-8 weeks old) were purchased from the Pasteur Institute of Iran. Throughout the study, the animals were kept under the controlled environmental conditions in standard cages (20 ± 2°C, 50-55% humidity, and 12/12 h light/dark cycle). When performing the experiment, all animals were given *ad libitum* access to safe water and rodent feed. Then, the study began by passing 7 days from the animal adaptation to environmental conditions. In the present study, the environmental conditions, use, and euthanasia of animals were considered in terms of the international ethical guidelines for laboratory animals [27] (ethical code obtained from Kurdistan University of Medical Sciences (IR.MUK.REC.1398.146)).

### 2.9. Experimental Design

The animals were randomly divided into 5 groups (6 rats per group) as follows:

- *Control Group (C)*. Only received distilled water (P.O.);
- *Nicotine Group (N)*. Only received nicotine (0.5 mg/kg/day, I.P.);
- *Treatment Group 1 (T1)*. Nicotine (0.5 mg/kg/day, I.P.) + AME (100 mg/kg/day, P.O.);
- *Treatment Group 2 (T2)*. Nicotine (0.5 mg/kg/day, I.P.) + AME (250 mg/kg/day, P.O.);
- *Treatment Group 3 (T3)*. Nicotine (0.5 mg/kg/day, I.P.) + AME (500 mg/kg/day, P.O.) [20, 26].

The study period was 21 days for all animals. Once at the beginning and once at the end of the study, the body weight of the animals was measured. At the end of the experimental period, the animals were anesthetized by intraperitoneal injection of ketamine-xylazine (100 and 10 mg/kg, respectively) and blood samples were collected from their right ventricle. The obtained blood samples were centrifuged for 15 min at 1000 g. Afterward, the attained serums were aliquoted and then stored at -20°C until processing. After euthanasia, each animal's liver was separated and then weighed. Subsequently, a piece of the left lobe of liver tissue (0.5 g) was immediately mixed with phosphate-buffered saline (1 : 9 ratio, 100 mM, and 7.4 pH) in a medium containing ice. Thereafter, it was homogenized by a homogenizer (OV5, Velp, Italy). Subsequently, tissue homogenates were centrifuged for 1 h at 5000 g at 4°C. Finally, the supernatant was stored at -20°C until the measurements of lipid peroxidation, enzymatic antioxidants, and proinflammatory cytokine levels. At the end of the study period, the ratio of liver to body weight was calculated [28].

### 2.10. Biochemical Assays

Serum activity levels of alanine aminotransferase (ALT), aspartate aminotransferase (AST), alkaline phosphatase (ALP), lactate dehydrogenase (LDH), and gamma-glutamyl transferase (GGT) and serum concentrations of total cholesterol (TC), triglyceride (TG), and high-density lipoprotein cholesterol (HDL-C) were determined using commercial kits (Pars Azmun, Iran) and the Hitachi 911 autoanalyzer [29]. LDL − C = TC − (HDL − C) − (0.2 × TG) and VLDL − C = 0.2 × TG formulas were also used to calculate low-density lipoprotein cholesterol (LDL-C) and very-low-density lipoprotein cholesterol (VLDL-C), respectively [30].

### 2.11. Determination of MDA Levels

Malondialdehyde (MDA) levels of tissue homogenates were measured in terms of the Buege and Aust method [31]. Briefly, 1 ml of reagent (15% trichloroacetic acid (TCA), 0.375% thiobarbituric acid (TBA), and 0.25 M hydrochloric acid (HCl)) was added to 500 *μ*l of 10% liver homogenate. This mixture was then placed in a bain-marie for 15 minutes at 95°C. After cooling, the mixture was centrifuged (10 min, 1000 g), and the obtained supernatant absorbance was determined at 535 nm. Finally, the MDA level was calculated by a 1.56 × 10^5^ M^−1^ cm^−1^ molar extinction coefficient. Notably, all the concentrations were expressed as nmol/g tissue.

### 2.12. Antioxidant Enzyme Activity Assay

In liver homogenates, the levels of superoxide dismutase (SOD), glutathione peroxidase (GPx), and catalase (CAT) were measured using colorimetric kits (ZellBio GmbH, Germany) in terms of the manufacturer's instructions. The basis of the determination of SOD activity was a conversion of O_2_^−^· to H_2_O_2_ under enzymatic conditions for 120 seconds at 420 nm. Next, CAT activity was measured based on its ability to decompose H_2_O_2_ within 1 minute at 405 nm. Also, GPx converted glutathione (GSH) to oxidized glutathione (GSSG). Hence, through the reaction of residual GSH, 5,5′-dithiobis-(2-nitrobenzoic acid) (DTNB) was used to determine GPx at 412 nm indirectly. All data were expressed as U/g tissue.

### 2.13. Proinflammatory Cytokine Assay

In liver homogenates, the levels of interleukin-1*β* (IL-1*β*, detection range: 15.6-1000 pg/ml), interleukin-6 (IL-6, detection range: 7.8-500 pg/ml), and tumor necrosis factor-*α* (TNF-*α*, detection range: 15.6-1000 pg/ml) were quantified using enzyme-linked immunosorbent assay (ELISA) kits (USCN, Wuhan, China) in terms of the manufacturer's protocols at 450 nm (microplate reader, Stat Fax 4200). The obtained results were then expressed in pg/ml.

### 2.14. Histopathological Assessments

The samples obtained from the liver right lobe were fixed for 48 h in 10% phosphate-buffered formalin. After the fixation, the samples were embedded in paraffin. Thereafter, 5 *μ*m thick sections of paraffin blocks were cut using a microtome and then stained with hematoxylin and eosin (H&E). Afterward, a semiquantitative scoring system was used to evaluate the histopathological criteria [32–34]. Accordingly, at least 10 fields of each liver section were examined using a light microscope by two pathologists who were blinded to the study (Nikon E100). Then, each criterion was scored from 0 to 3 (0 = none, 1 = mild, 2 = moderate, and 3 = severe). Accordingly, the evaluated criteria were as follows:

- Hydropic degeneration of hepatocytes
- Kupffer cell proliferation
- Central vein congestion and dilation of sinusoids

### 2.15. Statistical Analysis

The study data were reported as mean ± SEM. One-way analysis of variance (one-way ANOVA) followed by Tukey's post hoc test was performed to analyze the parametric data. Moreover, liver histopathological scores were assessed by the Kruskal–Wallis test. In this regard, if the Kruskal–Wallis test was significant, the Mann–Whitney test was then used to perform pairwise comparisons. All graphs and statistical analyses were performed by GraphPad Prism (Version 7.03) software (GraphPad Software, San Diego, California, USA). *P* value < 0.05 was considered the statistical significance level.

## 3. Results

### 3.1. Phenols, Flavonoids, and IC50

Table 1 depicts the total phenolic and flavonoid contents as well as IC50 of AME.

### 3.2. DPPH Radical Scavenging Activity

**Figure 1:**
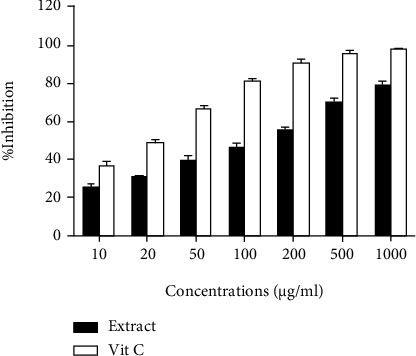
Inhibition percentage of DPPH radicals by AME and vitamin C at different concentrations. Each column represents the mean ± SEM (*n* = 3).

As shown in Figure 1, the inhibition percentage of DPPH radicals was dose-dependent by AME and vitamin C; therefore, in the range of concentrations of 10 to 1000 *μ*g/ml, the inhibitory percentage of the extract increased from 18.94 ± 0.97 up to 77.87 ± 2.07, and for vitamin C, it increased from 36.86 ± 1.82 up to 98.69 ± 0.40.

### 3.3. LD50 Test

During the 14 days of the acute toxicity test, the animal's death and body weight changes were not observed. LD50 was higher than the oral administration of 2000 mg extract/kg.

### 3.4. Body Weight

**Figure 2:**
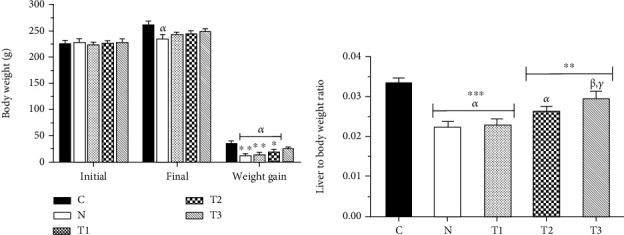
Body weight (a) and liver-to-body weight ratio (b) in the groups. Each column represents the mean ± SEM (*n* = 6). ^∗^*P* < 0.05, ^∗∗^*P* < 0.01, and ^∗∗∗^*P* < 0.001. *α*, *β*, and *γ* compared to the C, N, and T1 groups, respectively.

At the end of the study, the body weight of the N group (235.96 ± 7.04 g) has significantly decreased (*P* < 0.05) compared to that of the C group (262.92 ± 5.99 g). The highest increase in body weight gain was observed in the C group (36.06 ± 3.98 g), and the lowest one was for the N group (12.41 ± 2.41 g). Among the AME-treated groups, the T1 group had the lowest (14.57 ± 2.69 g) and the T3 group had the highest (24.74 ± 2.67 g) increase in weight gain. A significant decrease was also observed in body weight gain among the N, T1, and T2 groups (19.04 ± 5.78 g) compared to the control group (C) (*P* < 0.05). Notably, there was no significant difference between the T3 and C groups (*P* > 0.05) (Figure 2(a)).

Furthermore, the lowest total food consumption was in the N group and the highest was observed in the control group. In this regard, the lowest average water consumption during the study period was in the N group, and this difference was substantial when compared to the other groups (*P* < 0.05) (Table 2).

### 3.5. Liver-to-Body Weight Ratio

As shown in Figure 2(b), the liver-to-body weight ratio has significantly decreased in the N (0.022 ± 0.001 g), T1 (0.023 ± 0.001 g), and T2 (0.027 ± 0.001 g) groups compared to the C group (0.034 ± 0.001 g) (*P* < 0.05). Moreover, there was a significant increase in terms of the liver-to-body weight ratio in the T3 group (0.030 ± 0.002 g) when the extract was administered at a dose of 500 mg/kg/day compared to the N and T1 groups (*P* < 0.05). In addition, no significant difference was observed in the liver-to-body weight ratio between the T3 and C groups (*P* > 0.05).

### 3.6. Liver Function Enzymes

Moreover, nicotine intake (the N group) significantly increased the serum activities of ALT, AST, ALP, GGT, and LDH compared to the control group (C group). Administration of this extract has also decreased the serum activities of liver enzymes. Among the AME-treated groups, the most significant decrease in the serum activities of enzymes was observed in the animals receiving the extract at a dose of 500 mg/kg/day (the T3 group) compared to the N group (Table 3).

### 3.7. Lipid Profile

As shown in Table 3, the serum levels of TC and LDL-C have significantly increased in the N group compared to the C group (*P* < 0.05). Moreover, serum concentrations of TG, HDL-C, and VLDL-C have significantly decreased (*P* < 0.05). Lipid profiles (TC, TG, HDL-C, LDL-C, and VLDL-C) of the T3 group have also shown a significant difference compared to those of the N and T1 groups (*P* < 0.05). In addition, TG, HDL-C, and VLDL-C levels had no significant difference in the group receiving the extract at a dose of 250 mg/kg/day (the T2 group) compared to the other groups (*P* > 0.05).

### 3.8. Hepatic Oxidative Stress Parameters

As shown in Table 4, MDA levels have increased significantly. Moreover, SOD, CAT, and GPx levels showed a significant decrease in liver tissue of the N group compared to the C group (*P* < 0.05). It is noteworthy that MDA, SOD, CAT, and GPx levels of liver tissue in AME-treated groups were dose-dependent, and accordingly, the most significant efficacy of the extract was in the T3 group compared to the N group.

### 3.9. Proinflammatory Cytokine Levels

**Figure 3:**
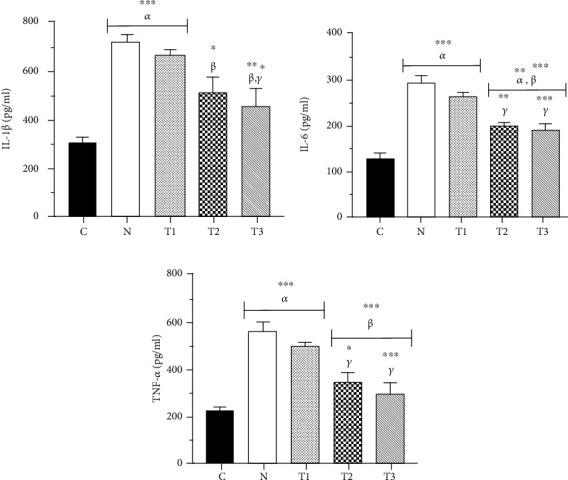
Concentrations of IL-1*β* (a), IL-6 (b), and TNF-*α* (c) in the liver of rats. Each column represents the mean ± SEM (*n* = 6). ^∗^*P* < 0.05, ^∗∗^*P* < 0.01, and ^∗∗∗^*P* < 0.001. *α*, *β*, and *γ* compared to the C, N, and T1 groups, respectively.

The level of IL-1*β* has significantly increased in the N (725.84 ± 25.70 pg/ml) and T1 (674.91 ± 17.78 pg/ml) groups compared to the C group (311.31 ± 15.50 pg/ml) (*P* < 0.05). In addition, liver tissue IL-1*β* concentration showed a significant decrease in the T3 group (454.78 ± 75.58 pg/ml), in which the extract with the dose of 500 mg/kg/day was administered, compared to the T1 and N groups (*P* < 0.05). Also, the level of IL-1*β* has significantly decreased in the T2 group (512.37 ± 64.19 pg/ml) compared to the nicotine-receiving group (N group, *P* < 0.05) (Figure 3(a)).

As shown in Figure 3(b), the liver IL-6 levels have significantly increased in the animals of the N (290.82 ± 12.89 pg/ml) and T1 (258.69 ± 7.36 pg/ml) groups compared to the C group (123.97 ± 13.52 pg/ml) (*P* < 0.05). Furthermore, IL-6 concentration in the T3 (184.93 ± 13.99 pg/ml) and T2 (194.82 ± 5.97 pg/ml) groups has significantly decreased compared to that in the animals of the T1 and N groups, which showed a significant increase compared to the C group (*P* < 0.05).

The TNF-*α* concentration in the liver tissue of the T2 (342.72 ± 40.23 pg/ml) group receiving the extract at a dose of 250 mg/kg/day showed a significant decrease compared to that of the T1 (495.14 ± 10.60 pg/ml) and N (558.79 ± 31.82 pg/ml) groups (*P* < 0.05). Correspondingly, this decrease was also more pronounced when the extract was administered at the dose of 500 mg/kg/day in the T3 group (294.98 ± 40.77 pg/ml) (*P* < 0.05). The level of liver TNF-*α* in animals of the N and T1 groups has significantly increased compared to that of the C group (224.97 ± 12.07 pg/ml) (*P* < 0.05) (Figure 3(c)).

### 3.10. Liver Histopathology

**Figure 4:**
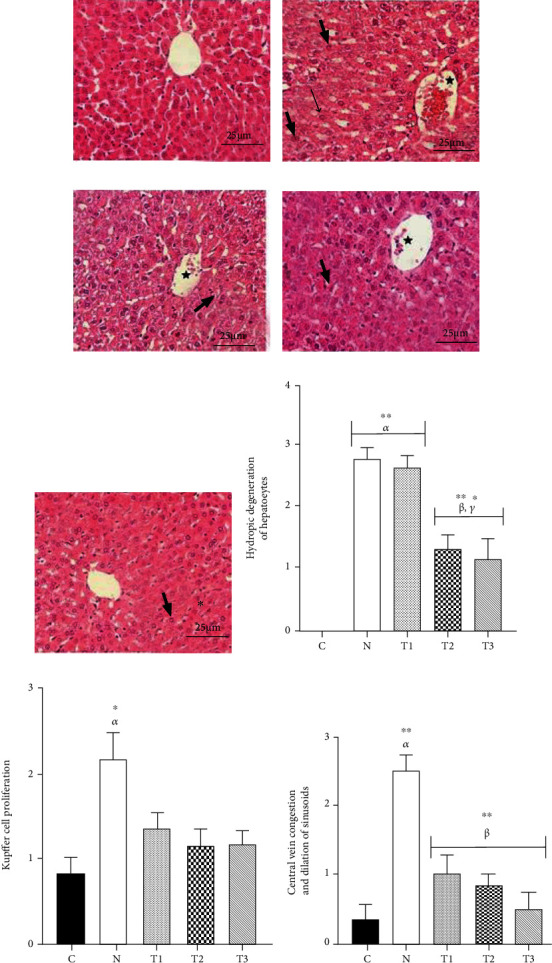
Microscopic view of animal liver tissue in the groups (H&E staining, ×400 magnification). C group: the structure of the liver is normal (a). N group: severe hydropic degeneration of hepatocytes (thick arrows), central vein congestion (star), and Kupffer cell proliferation (narrow arrow) are shown (b). T1 group: severe hydropic degeneration of hepatocytes (thick arrow) and central vein congestion (star) are observable (c). T2 group: mild-to-moderate hydropic degeneration of hepatocytes (thick arrow) and central vein congestion (star) are seen (d). T3 group: mild hydropic degeneration of hepatocytes (thick arrow) is shown (e). Scores of hydropic vacuolation of hepatocytes (f), Kupffer cell proliferation (g), and central vein congestion and dilation of sinusoids (h) are displayed. Each column represents the mean ± SEM (*n* = 6). ^∗^*P* < 0.05 and ^∗∗^*P* < 0.01. *α*, *β*, and *γ* compared to the C, N, and T1 groups, respectively.

Nicotine administration was associated with some severe pathological changes in liver tissue. In addition, the criterion of hydropic degeneration of hepatocytes has significantly increased in the N (2.83 ± 0.17) and T1 (2.67 ± 0.21) groups compared to the C group (0.0 ± 0.0) (*P* < 0.05). Accordingly, when the extract was administered at doses of 250 and 500 mg/kg/day, the scores of this criterion have significantly decreased in the T2 (1.33 ± 0.21) and T3 (1.17 ± 0.31) groups compared to the N group (*P* < 0.05), respectively. This decrease was also repeated in the T2 and T3 groups compared to the T1 group (*P* < 0.05). The criterion scores of hydropic degenerations of hepatocytes have also increased in the T2 and T3 groups compared to the C group; however, it was not significant (*P* > 0.05) (Figure 4(f)).

As shown in Figure 4(g), the Kupffer cell proliferation criterion has significantly increased in animals of the N group (2.17 ± 0.31) that only received nicotine compared to the C group (0.83 ± 0.17). The score of this criterion has also decreased in the AME-treated groups compared to the N group and increased as well compared to the C group; however, no significant differences were observed in this regard (*P* > 0.05).

The criteria of central vein congestion and dilation of sinusoids have significantly increased in the N group (2.50 ± 0.22) compared to the C group (0.33 ± 0.21) (*P* < 0.05). These scores have significantly decreased in the T3 (0.50 ± 0.22), T2 (0.83 ± 0.17), and T1 (1.00 ± 0.26) groups compared to the N group (*P* < 0.05) (Figure 4(h)).

The histopathological changes of liver tissue of the animals in the studied groups are microscopically shown in Figures 4(a)–4(e).

## 4. Discussion

Plants are rich sources of biologically active compounds with wide molecular diversities. These sources are known for the development of new drugs [35–37]. In this regard, polyphenolic compounds (phenols and flavonoids) are among the most effective compounds. Although they facilitate the catch of free radicals, they demonstrate anti-inflammatory and antimicrobial properties [38]. In the present study, the total phenolic and flavonoid contents of AME were determined, which were consistent with the results of the other studies [13, 39].

In recent years, the wide range of the effects of free radicals has interested the attention of scientists, and as a result, it has been proven that free radicals play a remarkable role in the pathogenesis of many diseases such as cancer [40]. Antioxidants are also known as a noticeable part of plant compounds that can delay or stop lipid oxidation as well as the other molecules via inhibiting the initiation or the release of oxidation chain reactions [16, 41, 42]. The measurement of the inhibitory activity of DPPH radicals was performed based on the use of the stable DPPH free radicals, which have significantly decreased when facing the antioxidants. Therefore, it can be used to evaluate the antiradical activities of food and plant extracts [43]. In the present study, the IC50 of AME was measured to be less than 500 *μ*g/ml, while the studied concentrations were between 0 and 1000 *μ*g/ml in the DPPH test. Therefore, the IC50 lower than the median indicates the high antioxidant power of alfalfa in inhibiting free radicals. In this regard, the high inhibitory activity of the extract probably resulted from polyphenolic compounds [44].

In experimental toxicology studies, body weight and the liver-to-body weight ratio are vital and readily available indices when facing oxidative stress [45]. In the current study, body weight was significantly decreased by nicotine administration in the control group. This decrease can probably be due to the nicotine effect on reducing calorie intake through increasing the release of neurotransmitters such as dopamine and serotonin [46]. Moreover, administration of AME increased weight gain in animals treated with nicotine. Although the action mechanism of alfalfa during this process has not been understood yet, alfalfa appears to have a central slight antidopaminergic action [47]. Thus, considering this mechanism, it was shown that it can control the harmful effect of nicotine on reducing calorie intake. Furthermore, the nicotine-induced liver-to-body weight ratio showed a significant decrease that could be either due to its effect on body weight or due to liver damage [48]. The extract improved the liver-to-body weight ratio; therefore, no difference was observed between the control and T3 groups (dose, 500 mg/kg/day). This improvement may be induced by the antidopaminergic and antioxidant effects of the plant [49].

The liver plays a key role in the metabolism of drugs, foods, and many compounds, so changing its function endangers a person's health [50]. Liver damage, acute or chronic, could eventually lead to the increased serum activities of some liver enzymes such as ALT, AST, ALP, GGT, and LDH [51]. In liver cells, the main AST activity is mitochondrial; however, ALT is exclusively found in the cytoplasm [52]. Although ALP, GGT, and LDH are found in many tissues, some previous studies have shown that the most common reason for these enzymes' increase is a defect in liver function [53]. In the present study, nicotine administration has increased the serum activities of ALT, AST, ALP, GGT, and LDH enzymes, which is consistent with the findings of the other reports [54, 55]. It is possible that nicotine may destroy the membrane of liver cells by increasing the production of free radicals and then cause cellular leakage, which could eventually lead to the release of cytoplasmic and mitochondrial enzymes into the bloodstream [56]. Subsequently, the administration of alfalfa extract has decreased the serum activities of liver enzymes in nicotine-treated animals. Accordingly, this result is justified because alfalfa, with its various vitamins and polyphenol compounds, has a great ability in trapping the produced free radicals by nicotine administration [57]. As a result, nicotine-induced liver damage would be inhibited and serum activities of enzymes would be decreased.

Several studies have shown a link between heart diseases and lipid profile changes [58, 59]. In our study, nicotine administration was shown to be associated with increased serum levels of TC and LDL-C. In addition, TG, HDL-C, and VLDL-C levels have also decreased. Apparently, the increase in TC levels was due to the increased cholesterol synthesis via inducing hepatic HMG-CoA reductase activity in the path of cholesterol biosynthesis [60]. Moreover, HDL-C transports cholesterol to the liver for breakdown so that its reduction would lead to an increase in TC [61]. The increase in LDL-C was indicated to be related to the alkaloid properties of nicotine. Correspondingly, this feature enhances the entry of lipoprotein particles into liver cells, which consequently leads to increased LDL-C by changing the smooth endoplasmic reticulum [62]. The mechanism of TG and VLDL-C reduction due to nicotine administration is still unclear. Although VLDL particles are known as the main carriers of TG transmission in the blood, when it decreases, TG would be declined as well [63]. In the present study, the administration of AME for the nicotine-treated animals normalized the lipid profile. Although the action mechanism of alfalfa is still unknown in this regard, the intervention in gene expression and neutralization of the nicotine alkaloid effects through the involvement in the biosynthesis cycle of lipids can be mentioned [64].

MDA is used as an important indicator in the diagnosis of the induced stress within the target tissue [65]. In our study, we found that nicotine administration increases MDA in liver tissue. Moreover, by producing ROS, nicotine appears to induce oxidative stress, which eventually leads to lipid peroxidation [66]. Subsequently, the body uses SOD, CAT, and GPx antioxidants to deal with the produced ROS.

In harmony with these results, Taysi et al. reported that nicotine performance at a dose of 0.5 mg/kg intraperitoneally for 21 days caused a significant increase in malondialdehyde, nitric oxide, and glutathione reductase (GR), as well as a decrease in GPx in liver tissue [26]. In addition, Helen et al. in their study announced a significant increase in the concentration of total superoxide scavenger activity, TBA reactive substances (TBARS), hydroperoxides, and conjugated dienes in the liver tissue as lipid peroxidation products [67]. Another similar study conducted by El-Sokkary and coworkers explained the nicotine increase in malondialdehyde and the decrease in SOD and GSH levels in the liver. Also, as pathological changes, the occurrence of focal cell necrosis indicates the death of a significant proportion of liver cells. Fatty change of the liver reflects an imbalance of the production, utilization, and mobilization of lipids by liver cells and is an indicator of nonlethal injury. The histopathological changes in the liver of nicotine-treated rats suggest that additional specific pathogenic pathways may be involved in nicotine hepatotoxicity [68]. Hence, these antioxidants have decreased in animals treated with nicotine. In addition, in the present study, AME administration has decreased MDA levels and increased SOD, CAT, and GPx antioxidant levels. These findings were consistent with the pathological evidence.

Apparently, alfalfa has various vitamins (such as vitamins C and E), polyphenolic compounds (such as flavonoids and phenols), some types of minerals (such as phosphorus and calcium), some types of phytosterols, and other antioxidant compounds [69, 70]. Therefore, these compounds trap free radicals and also decrease the stress and oxidative effects of nicotine.

One of the most important indices of tissue damage is inflammation [71]. In this regard, the main advantageous indicators for measuring tissue inflammation are proinflammatory cytokines (IL-1*β*, IL-6, and TNF-*α*) [72, 73]. Accordingly, these cytokines are secreted through the immune system cells involved in the inflammation (e.g., macrophages, monocytes, and regulatory T cells) [74, 75]. In the present study, nicotine administration has increased levels of proinflammatory cytokines (IL-1*β*, IL-6, and TNF-*α*). Also, it seems that the induction of nicotine-induced oxidative stress and the production of free radicals have consequently resulted in cell destruction and the invocation of immune cells to the liver, as the target tissue of nicotine [76, 77]. Therefore, because of the inflammation caused by cellular and tissue destructions, to create an inflammatory cascade, immune cells have produced proinflammatory cytokines (IL-1*β*, IL-6, and TNF-*α*) [78]. In our study, the administration of the extract has decreased the levels of these cytokines; therefore, the highest reduction was observed in the T3 group. It is possible that alfalfa decreased the damage of liver cells via removing free radicals caused by nicotine, and thus, the development of inflammation has consequently reduced [20].

One of the characteristics of working with laboratory animals is access to target tissues in histopathological studies [79]. In previous studies, nicotine administration has been shown to be associated with the infiltration of inflammatory cells and liver damage [80, 81]. In the present study, in conjunction with some other studies, the proliferation of Kupffer cells, hydropic degeneration, and central vein congestion were observed in the liver tissue. It was hypothesized that nicotine metabolism may have caused these histopathological changes through cytochrome P450 enzymes and the production of free radicals [82]. In the present study, the administration of the extract controlled histopathological changes and reduced lesions. This result could be considered to be in line with the antioxidant power of the extract in response to the mechanism of nicotine-induced tissue damage by free radicals [83]. In another study, the neuroprotective and behavioral effects of alfalfa ethanolic extract against nicotine were reported. Therefore, we can point to the positive systemic function of alfalfa extracts in different ways of administration to various nicotine target tissues [84].

## 5. Conclusions

In conclusion, the presence of polyphenolic compounds, as well as the IC50 value below the median concentration, showed the high antioxidant power of AME. Therefore, this plant can be considered an important antiradical in complementary medicine more than before. In this study, nicotine has resulted in weight loss; decreased liver-to-body weight ratios; increased serum activities of liver enzymes (ALT, AST, ALP, GGT, and LDH); altered lipid profiles; increased liver MDA levels; decreased levels of SOD, CAT, and GPx antioxidants in liver tissue; and increased hepatic levels of proinflammatory cytokines (IL-1*β*, IL-6, and TNF-*α*), and it resulted in histopathological changes in the liver tissue. This study showed that AME with phenolic compounds has a high antioxidant power, which can control the mentioned effects of nicotine. This control was revealed through the laboratory findings of the present study. Trapping nicotine-induced free radicals, boosting the body's immune and antioxidant systems, and affecting metabolic pathways are some of the functional reasons for alfalfa. In any case, it should be noted that studies in the experimental phase will be an introduction to the process of supplementary studies.

Since alfalfa has application both in the food industry and in preventive medicine to reduce the risk of toxicity such as liver damage (an important organ in detoxification as it is exposed to all of the toxicants), heart disease, and metabolic problems, alfalfa can be used as a supplement to suppress oxidative stress that is growing in our lives due to widespread application in all aspects. Moreover, recommending the use of antioxidant compounds, for example, alfalfa, in smokers to prevent the effects of nicotine oxidative stress can be another clinical application of the study.

## Figures and Tables

**Table 1:** Total phenols, total flavonoids, and IC50 of AME.

Sample	Total phenolic content (mg GAE/g extract)	Total flavonoid content (mg QE/g extract)	IC50 (*μ*g/ml)
Alfalfa methanolic extract (AME)	51.68 ± 0.62	18.55 ± 1.01	350.91 ± 16.46

**Table 2:** Food and water consumption in the groups in the period study.

Parameters			Groups		
	C	N	T1	T2	T3
Food consumption (g)	504.2 ± 12.39	340.2 ± 15.72*α*^∗∗∗^	470.7 ± 18.36*α*^∗^, *β*^∗∗^	485.1 ± 20.15*α*^∗^, *β*^∗∗^	485.5 ± 20.39*α*^∗^, *β*^∗∗∗^
Water intake (ml)	3376.4 ± 213.4	3160.5 ± 14.5*α*^∗∗^	3296.6 ± 24.6*β*^∗^	3365.8 ± 27.3*β*^∗^, *γ*^∗^	3371.2 ± 42.7*β*^∗∗^, *γ*^∗^

All values are expressed as mean ± SEM (*n* = 6). ^∗^*P* < 0.05, ^∗∗^*P* < 0.01, and ^∗∗∗^*P* < 0.001. *α*, *β*, *γ*, and *δ* compared to the C, N, T1, and T2 groups, respectively.

**Table 3:** Serum activities of liver enzymes and levels of lipid profiles in the groups.

Parameters	Groups				
	C	N	T1	T2	T3
ALT (U/l)	57.83 ± 2.32	111.30 ± 2.89*α*^∗∗∗^	102.70 ± 3.75*α*^∗∗∗^, *β*^∗^	83.50 ± 4.83*α*^∗∗∗^, *β*^∗∗∗^, *γ*^∗∗^	71.67 ± 3.96*β*^∗∗∗^, *γ*^∗∗∗^
AST (U/l)	103.80 ± 3.30	247.70 ± 13.46*α*^∗∗∗^	228.70 ± 11.39*α*^∗∗∗^	187.30 ± 6.71*α*^∗∗∗^, *β*^∗∗∗^, *γ*^∗^	140.20 ± 5.30*β*^∗∗∗^, *γ*^∗∗∗^, *δ*^∗∗^
ALP (U/l)	297.20 ± 10.93	453.70 ± 12.57*α*^∗∗∗^	429.20 ± 4.32*α*^∗∗∗^	391.80 ± 9.03*α*^∗∗∗^, *β*^∗∗∗^	342.20 ± 8.39*α*^∗^, *β*^∗∗∗^, *γ*^∗∗∗^, *δ*^∗∗^
GGT (U/l)	6.83 ± 0.60	17.17 ± 0.95*α*^∗∗∗^	15.33 ± 0.76*α*^∗∗∗^	11.50 ± 0.96*α*^∗∗^, *β*^∗∗^, *γ*^∗^	9.83 ± 1.14*β*^∗∗∗^, *γ*^∗∗^
LDH (U/l)	785.70 ± 18.15	1182.0 ± 40.31*α*^∗∗∗^	1063.0 ± 42.80*α*^∗∗∗^	897.70 ± 15.38*β*^∗∗∗^, *γ*^∗∗^	835.20 ± 19.08*β*^∗∗∗^, *γ*^∗∗∗^
TC (mg/dl)	105.20 ± 5.42	149.80 ± 7.88*α*^∗∗∗^	141.0 ± 3.50*α*^∗∗∗^	126.50 ± 2.72*β*^∗^	114.30 ± 5.68*β*^∗∗∗^, *γ*^∗^
TG (mg/dl)	93.67 ± 4.06	78.0 ± 2.13*α*^∗∗^	80.50 ± 2.32*α*^∗^	86.33 ± 1.63	91.83 ± 2.52*β*^∗∗^, *γ*^∗^
HDL-C (mg/dl)	55.83 ± 2.27	36.17 ± 1.74*α*^∗∗∗^	39.83 ± 2.87*α*^∗∗^	46.67 ± 3.48	51.33 ± 2.58*β*^∗∗^, *γ*^∗^
LDL-C (mg/dl)	30.60 ± 5.44	98.07 ± 8.32*α*^∗∗∗^	85.07 ± 1.72*α*^∗∗∗^	62.57 ± 6.16*α*^∗^, *β*^∗∗^	44.63 ± 7.92*β*^∗∗∗^, *γ*^∗∗^
VLDL-C (mg/dl)	18.73 ± 0.81	15.60 ± 0.43*α*^∗∗^	16.10 ± 0.46*α*^∗^	17.27 ± 0.33	18.37 ± 0.50*β*^∗∗^, *γ*^∗^

All values are expressed as mean ± SEM (*n* = 6). ^∗^*P* < 0.05, ^∗∗^*P* < 0.01, and ^∗∗∗^*P* < 0.001. *α*, *β*, *γ*, and *δ* compared to the C, N, T1, and T2 groups, respectively.

**Table 4:** Levels of lipid peroxidation and enzymatic antioxidants in the liver of animals.

Parameters	Groups				
	C	N	T1	T2	T3
MDA (nmol/g tissue)	4.79 ± 0.33	10.22 ± 0.57*α*^∗∗∗^	9.09 ± 0.45*α*^∗∗∗^	6.91 ± 0.64*α*^∗^, *β*^∗∗∗^, *γ*^∗^	5.99 ± 0.39*β*^∗∗∗^, *γ*^∗∗^
SOD (U/g tissue)	37.34 ± 3.44	16.69 ± 0.85*α*^∗∗∗^	19.79 ± 1.14*α*^∗∗∗^	27.03 ± 0.82*α*^∗^, *β*^∗^	30.16 ± 2.57*β*^∗∗∗^, *γ*^∗^
CAT (U/g tissue)	31.33 ± 1.26	12.45 ± 0.77*α*^∗∗∗^	15.69 ± 0.84*α*^∗∗∗^	19.92 ± 1.15*α*^∗∗∗^, *β*^∗∗∗^	26.65 ± 1.56*β*^∗∗∗^, *γ*^∗∗∗^, *δ*^∗∗^
GPx (U/g tissue)	230.80 ± 23.82	112.40 ± 10.91*α*^∗∗^	124.30 ± 15.20*α*^∗∗^	159.80 ± 19.98	195.30 ± 25.52*β*^∗^

All values are expressed as mean ± SEM (*n* = 6). ^∗^*P* < 0.05, ^∗∗^*P* < 0.01, and ^∗∗∗^*P* < 0.001. *α*, *β*, *γ*, and *δ* compared to the C, N, T1, and T2 groups, respectively.

## Data Availability

The data used to support the findings of this study are available from the corresponding author upon request.

## References

[1] Li H., Xie K., Yu W. (2016). Nicotine dehydrogenase complexed with 6-hydroxypseudooxynicotine oxidase involved in the hybrid nicotine-degrading pathway in Agrobacterium tumefaciens S33. *Applied and Environmental Microbiology*.

[2] Wu Y., Song P., Zhang W. (2015). Activation of AMPK*α*2 in adipocytes is essential for nicotine-induced insulin resistance _in vivo_. *Nature Medicine*.

[3] Ateyya H., Nader M. A., Attia G. M., el-Sherbeeny N. A. (2017). Influence of alpha-lipoic acid on nicotine-induced lung and liver damage in experimental rats. *Canadian Journal of Physiology and Pharmacology*.

[4] Mosbah R., Yousef M. I., Mantovani A. (2015). Nicotine-induced reproductive toxicity, oxidative damage, histological changes and haematotoxicity in male rats: the protective effects of green tea extract. *Experimental and Toxicologic Pathology*.

[5] Benowitz N. L., Hukkanen J., Jacob P. (2009). Nicotine chemistry, metabolism, kinetics and biomarkers. *Handbook of Experimental Pharmacology*.

[6] Conceição E. P., Peixoto-Silva N., Pinheiro C. R., Oliveira E., Moura E. G., Lisboa P. C. (2015). Maternal nicotine exposure leads to higher liver oxidative stress and steatosis in adult rat offspring. *Food and Chemical Toxicology*.

[7] Baykalir B. G., Arslan A. S., Mutlu S. I. (2021). The protective effect of chrysin against carbon tetrachloride-induced kidney and liver tissue damage in rats. *International Journal for Vitamin and Nutrition Research*.

[8] Zafra-Rojas Q. Y., González-Martínez B. E., Cruz-Cansino N. . S. (2020). Effect of ultrasound on in vitro bioaccessibility of phenolic compounds and antioxidant capacity of blackberry (Rubus fruticosus) residues cv. Tupy. *Plant Foods for Human Nutrition*.

[9] Li S., Tan H. Y., Wang N. (2015). The role of oxidative stress and antioxidants in liver diseases. *International Journal of Molecular Sciences*.

[10] Rietjens I. M., Louisse J., Beekmann K. (2017). The potential health effects of dietary phytoestrogens. *British Journal of Pharmacology*.

[11] Asensi-Fabado M. A., Munné-Bosch S. (2010). Vitamins in plants: occurrence, biosynthesis and antioxidant function. *Trends in Plant Science*.

[12] Soto-Zarazúa M. G., Bah M., Costa A. S. G. (2017). Nutraceutical potential of new alfalfa (Medicago sativa) ingredients for beverage preparations. *Journal of Medicinal Food*.

[13] Mirzaei A., Delaviz H., Mirzaei M., Tolooei M. (2015). The effects of Medicago sativa and Allium porrum on iron overload in rats. *Global Journal of Health Science*.

[14] Shenstone E., Lippman Z., Van Eck J. (2020). A review of nutritional properties and health benefits of Physalis species. *Plant Foods for Human Nutrition*.

[15] Berenjian S., Raeeszadeh M. (2016). Prescription of antibiotics before and after surgery at the surgical wards of Isfahan Amiralmomenin Hospital compliance with the standard guidelines. *Health Research Journal*.

[16] Raeeszadeh M., Fallah M. (2018). The comparison of the effect of Origanum vulgar aqueous extract and vitamin C on the control of cadmium chloride damage in testicular tissue in male rats. *Journal of Babol University of Medical Sciences*.

[17] Raeeszadeh M., Moradi M., Ayar P., Akbari A. (2021). The antioxidant effect of Medicago sativa L. (alfalfa) ethanolic extract against mercury chloride (HgCl_2_) toxicity in rat liver and kidney: an in vitro and in vivo study. *Evidence-based Complementary and Alternative Medicine*.

[18] Truong D.-H., Nguyen D. H., Ta N. T. A., Bui A. V., Do T. H., Nguyen H. C. (2019). Evaluation of the use of different solvents for phytochemical constituents, antioxidants, and in vitro anti-inflammatory activities of Severinia buxifolia. *Journal of Food Quality*.

[19] Seida A., el-Hefnawy H., Abou-Hussein D., Mokhtar F. A., Abdel-Naim A. (2015). Evaluation of Medicago sativa L. sprouts as antihyperlipidemic and antihyperglycemic agent. *Pakistan Journal of Pharmaceutical Sciences*.

[20] Al-Dosari M. S. (2012). In vitroandin vivoantioxidant activity of alfalfa (Medicago sativaL.) on carbon tetrachloride intoxicated rats. *Chinese Medicine*.

[21] Albayrak S., Aksoy A., Yurtseven L., Yaşar A. (2014). A comparative study on phenolic components and biological activity of someSeneciospecies in Turkey. *Journal of Pharmacy and Pharmacology*.

[22] Chang C.-C., Yang M. H., Wen H. M., Chern J. C. (2002). Estimation of total flavonoid content in propolis by two complementary colometric methods. *Journal of Food and Drug Analysis*.

[23] Ekin S., Bayramoglu M., Goktasoglu A., Ozgokce F., Kiziltas H. (2017). Antioxidant activity of aqueous and ethanol extracts of Crataegus meyeri Pojark leaves and contents of vitamin, trace element. *Journal of the Chilean Chemical Society*.

[24] Oliveira I., Coelho V., Baltasar R., Pereira J. A., Baptista P. (2009). Scavenging capacity of strawberry tree (*Arbutus unedo* L.) leaves on free radicals. *Food and Chemical Toxicology*.

[25] OECD, O. (2001). *Guideline for the testing of chemicals. Acute oral toxicity - acute toxic class method: test no-423*.

[26] Taysi S., Gumustekin K., Demircan B. (2010). Hippophae rhamnoides attenuates nicotine-induced oxidative stress in rat liver. *Pharmaceutical Biology*.

[27] Council, N.R. (2010). *Guide for the Care and Use of Laboratory Animals*.

[28] Liu J.-P., Zou W. L., Chen S. J. (2016). Effects of different diets on intestinal microbiota and nonalcoholic fatty liver disease development. *World Journal of Gastroenterology*.

[29] Abbasalipourkabir R., Moradi H., Zarei S. (2015). Toxicity of zinc oxide nanoparticles on adult male Wistar rats. *Food and Chemical Toxicology*.

[30] Raeeszadeh M., Beheshtipour J. (2018). Letter to the editor. *Naunyn-Schmiedeberg's Archives of Pharmacology*.

[31] Buege J. A., Aust S. D. (1978). [30] Microsomal lipid peroxidation. *Methods in Enzymology*.

[32] Özdemir-Kumral Z. N., Özbeyli D., Özdemir A. F. (2017). Protective effect of nicotine on sepsis-induced oxidative multiorgan damage: role of neutrophils. *Nicotine & Tobacco Research*.

[33] Beheshtipour J., Akradi L., Raeeszadeh M. (2018). The effect of aqueous extract of grapevine leaf (Vitis vinifera) on pathologic feature of the pancreas in type-1 experimental diabetes: a different approach to medicinal plants. *Scientific Journal of Kurdistan University of Medical Sciences*.

[34] Raeeszadeh M., Ahmadi E., Shafiee M. (2016). Identification of the antibiotic resistance patterns in bacteria isolated from urinary trac infections in pations admited to Shahid Ghazi Hospital-Sansandaj in the first 6 month of 2014. *Journal of Iran University of Medical Sciences*.

[35] Altemimi A., Lakhssassi N., Baharlouei A., Watson D., Lightfoot D. (2017). Phytochemicals: extraction, isolation, and identification of bioactive compounds from plant extracts. *Plants*.

[36] Qaderi Forough M., Raeeszadeh M., Amiri A. (2017). Dose-response changes of Brassica oleracea var. italica hydroalcholic extract in the control of oxidative stress by induction of diazinon on the cells of testicular tissue in male adult rat. *Journal of Rafsanjan University of Medical Sciences*.

[37] Paryab M., Raeeszadeh M. (2016). The study of the rate and reasons of medical herb use by the patients visiting the specialized treatment centers in Fars province in 2014. *Community Health Journal*.

[38] Tungmunnithum D., Thongboonyou A., Pholboon A., Yangsabai A. (2018). Flavonoids and other phenolic compounds from medicinal plants for pharmaceutical and medical aspects: an overview. *Medicine*.

[39] Chen S., Li X., Liu X. (2020). Investigation of chemical composition, antioxidant activity, and the effects of alfalfa flavonoids on growth performance. *Oxidative medicine and cellular longevity*.

[40] Ríos-Arrabal S., Artacho-Cordón F., León J. (2013). Involvement of free radicals in breast cancer. *Springerplus*.

[41] Prisacaru A. E. (2016). Effect of antioxidants on polyunsaturated fatty acids - review. *Alimentaria*.

[42] Raeeszadeh M., Akbari A. (2021). The effects of broccoli and caraway extracts on serum oxidative markers, testicular structure and function, and sperm quality before and after sperm cryopreservation. *Cryobiology*.

[43] Hadadi Z., Nematzadeh G. A., Ghahari S. (2020). A study on the antioxidant and antimicrobial activities in the chloroformic and methanolic extracts of 6 important medicinal plants collected from north of Iran. *BMC chemistry*.

[44] Bustos A. S., Håkansson A., Linares-Pastén J. A., Penarrieta J. M., Nilsson L. (2018). Interaction between phenolic compounds and lipase: the influence of solubility and presence of particles in the IC50 value. *Journal of Food Science*.

[45] Campion S., Aubrecht J., Boekelheide K. (2013). The current status of biomarkers for predicting toxicity. *Expert Opinion on Drug Metabolism & Toxicology*.

[46] Stojakovic A., Espinosa E. P., Farhad O. T., Lutfy K. (2017). Effects of nicotine on homeostatic and hedonic components of food intake. *Journal of Endocrinology*.

[47] Veselinović T., Vernaleken I., Cumming P. (2018). Antidopaminergic medication in healthy subjects provokes subjective and objective mental impairments tightly correlated with perturbation of biogenic monoamine metabolism and prolactin secretion. *Neuropsychiatric Disease and Treatment*.

[48] Sinha-Hikim A. P., Sinha-Hikim I., Friedman T. C. (2017). Connection of nicotine to diet-induced obesity and non-alcoholic fatty liver disease: cellular and mechanistic insights. *Frontiers in Endocrinology*.

[49] Li J., Essemine J., Shang C. (2020). Combined proteomics and metabolism analysis unravels prominent roles of antioxidant system in the prevention of alfalfa (Medicago sativa L.) against salt stress. *International Journal of Molecular Sciences*.

[50] Gu X., Manautou J. E. (2012). Molecular mechanisms underlying chemical liver injury. *Expert Reviews in Molecular Medicine*.

[51] Malakouti M., Kataria A., Ali S. K., Schenker S. (2017). Elevated liver enzymes in asymptomatic patients–what should I do?. *Journal of Clinical and Translational Hepatology*.

[52] Zoppini G., Cacciatori V., Negri C. (2016). The aspartate aminotransferase-to-alanine aminotransferase ratio predicts all-cause and cardiovascular mortality in patients with type 2 diabetes. *Medicine*.

[53] Fernando S., Wijewickrama A., Gomes L. (2016). Patterns and causes of liver involvement in acute dengue infection. *BMC Infectious Diseases*.

[54] Wannamethee S. G., Shaper A. G. (2010). Cigarette smoking and serum liver enzymes: the role of alcohol and inflammation. *Annals of Clinical Biochemistry*.

[55] Yu C., Zhang Z., Liu Y. (2016). Toxicity of smokeless tobacco extract after 184-day repeated oral administration in rats. *International Journal of Environmental Research and Public Health*.

[56] El-Zayadi A.-R. (2006). Heavy smoking and liver. *World Journal of Gastroenterology: WJG*.

[57] Bora K. S., Sharma A. (2011). Phytochemical and pharmacological potential of Medicago sativa: a review. *Pharmaceutical Biology*.

[58] Sherpa L. Y., Deji, Stigum H. (2011). Lipid profile and its association with risk factors for coronary heart disease in the highlanders of Lhasa, Tibet. *High altitude medicine & biology*.

[59] Upadhyay R. K. (2015). Emerging risk biomarkers in cardiovascular diseases and disorders. *Journal of lipids*.

[60] Song Y., Zhang X., Chen W., Gao L. (2017). Cholesterol synthesis increased in the MMI-induced subclinical hypothyroidism mice model. *International Journal of Endocrinology*.

[61] Ali K. M., Wonnerth A., Huber K., Wojta J. (2012). Cardiovascular disease risk reduction by raising HDL cholesterol – current therapies and future opportunities. *British Journal of Pharmacology*.

[62] Gepner A. D., Piper M. E., Johnson H. M., Fiore M. C., Baker T. B., Stein J. H. (2011). Effects of smoking and smoking cessation on lipids and lipoproteins: outcomes from a randomized clinical trial. *American Heart Journal*.

[63] Mittendorfer B., Yoshino M., Patterson B. W., Klein S. (2016). VLDL triglyceride kinetics in lean, overweight, and obese men and women. *The Journal of Clinical Endocrinology & Metabolism*.

[64] Shi Y., Guo R., Wang X. (2014). The regulation of alfalfa saponin extract on key genes involved in hepatic cholesterol metabolism in hyperlipidemic rats. *PLoS One*.

[65] Ito F., Sono Y., Ito T. (2019). Measurement and clinical significance of lipid peroxidation as a biomarker of oxidative stress: oxidative stress in diabetes, atherosclerosis, and chronic inflammation. *Antioxidants*.

[66] Salahshoor M. R., Mahmoudian Z. G., Roshankhah S., Farokhi M., Jalili C. (2019). Harmine shows therapeutic activity on nicotine-induced liver failure in mice. *Histology and Histopathology*.

[67] Helen A., Krishnakumar K., Vijayammal P. L., Augusti K. T. (2000). Antioxidant effect of onion oil ( _Allium cepa_. Linn) on the damages induced by nicotine in rats as compared to alpha-tocopherol. *Toxicology Letters*.

[68] El-Sokkary G. H., Cuzzocrea S., Reiter R. J. (2007). Effect of chronic nicotine administration on the rat lung and liver: beneficial role of melatonin. *Toxicology*.

[69] Hong Y.-H., Wang S. C., Hsu C., Lin B. F., Kuo Y. H., Huang C. J. (2011). Phytoestrogenic compounds in alfalfa sprout (Medicago sativa) beyond coumestrol. *Journal of Agricultural and Food Chemistry*.

[70] Hwang J., Hodis H. N., Sevanian A. (2001). Soy and alfalfa phytoestrogen extracts become potent low-density lipoprotein antioxidants in the presence of acerola cherry extract. *Journal of Agricultural and Food Chemistry*.

[71] Chen L., Deng H., Cui H. (2018). Inflammatory responses and inflammation-associated diseases in organs. *Oncotarget*.

[72] Sproston N. R., Ashworth J. J. (2018). Role of C-reactive protein at sites of inflammation and infection. *Frontiers in Immunology*.

[73] Beheshtipour J., Raeeszadeh M. (2020). Evaluation of interleukin-10 and pro-inflammatory cytokine profile in calves naturally infected with neonatal calf diarrhea syndrome. *Archives of Razi Institute*.

[74] Raphael I., Nalawade S., Eagar T. N., Forsthuber T. G. (2015). T cell subsets and their signature cytokines in autoimmune and inflammatory diseases. *Cytokine*.

[75] Raeeszadeh M., Rezaee M., Akbari A., Khademi N. (2021). The comparison of the effect ofOriganum vulgareL. extract and vitamin C on the gentamycin-induced nephrotoxicity in rats. *Drug and Chemical Toxicology*.

[76] Mortaz E., Lazar Z., Koenderman L., Kraneveld A. D., Nijkamp F. P., Folkerts G. (2009). Cigarette smoke attenuates the production of cytokines by human plasmacytoid dendritic cells and enhances the release of IL-8 in response to TLR-9 stimulation. *Respiratory Research*.

[77] Strzelak A., Ratajczak A., Adamiec A., Feleszko W. (2018). Tobacco smoke induces and alters immune responses in the lung triggering inflammation, allergy, asthma and other lung diseases: a mechanistic review. *International Journal of Environmental Research and Public Health*.

[78] Kany S., Vollrath J. T., Relja B. (2019). Cytokines in inflammatory disease. *International Journal of Molecular Sciences*.

[79] Knoblaugh S. E., Hohl T. M., La Perle K. M. (2018). Pathology principles and practices for analysis of animal models. *ILAR Journal*.

[80] Jensen K., Nizamutdinov D., Guerrier M., Afroze S., Dostal D., Glaser S. (2012). General mechanisms of nicotine-induced fibrogenesis. *The FASEB Journal*.

[81] Salahshoor M., Mohamadian S., Kakabaraei S., Roshankhah S., Jalili C. (2016). Curcumin improves liver damage in male mice exposed to nicotine. *Journal of Traditional and Complementary Medicine*.

[82] Li D., Wang Y., Han K., Zhan C. G. (2010). Fundamental reaction pathways for cytochrome P450-catalyzed 5′-hydroxylation andN-demethylation of nicotine. *The Journal of Physical Chemistry B*.

[83] Xie Z., Huang J., Xu X., Jin Z. (2008). Antioxidant activity of peptides isolated from alfalfa leaf protein hydrolysate. *Food Chemistry*.

